# Transmission of Facial Expressions of Emotion Co-Evolved with Their Efficient Decoding in the Brain: Behavioral and Brain Evidence

**DOI:** 10.1371/journal.pone.0005625

**Published:** 2009-05-20

**Authors:** Philippe G. Schyns, Lucy S. Petro, Marie L. Smith

**Affiliations:** 1 Centre for Cognitive Neuroimaging (CCNi), University of Glasgow, Glasgow, United Kingdom; 2 Department of Psychology, University of Glasgow, Glasgow, United Kingdom; University of Granada, Spain

## Abstract

Competent social organisms will read the social signals of their peers. In primates, the face has evolved to transmit the organism's internal emotional state. Adaptive action suggests that the brain of the receiver has co-evolved to efficiently decode expression signals. Here, we review and integrate the evidence for this hypothesis. With a computational approach, we co-examined facial expressions as signals for data transmission and the brain as receiver and decoder of these signals. First, we show in a model observer that facial expressions form a lowly correlated signal set. Second, using time-resolved EEG data, we show how the brain uses spatial frequency information impinging on the retina to decorrelate expression categories. Between 140 to 200 ms following stimulus onset, independently in the left and right hemispheres, an information processing mechanism starts locally with encoding the eye, irrespective of expression, followed by a zooming out to processing the entire face, followed by a zooming back in to diagnostic features (e.g. the opened eyes in “fear”, the mouth in “happy”). A model categorizer demonstrates that at 200 ms, the left and right brain have represented enough information to predict behavioral categorization performance.

## Introduction

Primates use their faces to transmit facial expressions to their peers and communicate emotions. If the face evolved in part as a device to optimize transmission of facial expressions then the primate brain probably co-evolved as an efficient decoder of these signals: Competent social interaction implies a fast, on-line identification of facial expressions to enable adaptive actions. Here, we propose a computational theory addressing the two important questions of *how* and *when* the brain individuates facial expressions of emotion in order to categorize them quickly and accurately.

The manuscript is organized in two main parts. The first part outlines the evidence for the proposal that the face has evolved in part as a sophisticated system for signaling affects to peers. We conclude that the face communicates lowly correlated emotive signals. Turning to the receiver characteristics of these affects, we review the evidence that the brain comprises a sophisticated network of structures involved in the fast decoding and categorization of emotion signals, using inputs from low-level vision. These inputs represent information at different spatial scales analyzed across a bank of Spatial Frequency filters in early vision.

The second part of the paper develops our integrative computational account building from the data of Smith et al [1] and Schyns et al [2]. We also present new data to support the integration of the different parts of the research. Integrating the data of Smith et al [1] and Schyns et al [2] in a meta-analysis, we first show that the six basic categories of emotion (*happy*, *fear*, *surprise*, *disgust*, *anger*, *sadness* plus *neutral* [3]) constitute a set of lowly correlated signals for data transmission by the face. We then show that correct categorization behavior critically depends on using lowly correlated expression signals. The brain performs this decorrelation from facial information represented at different scales (i.e. across different spatial frequency bands) impinging on the observer's retina. Integrating the data of Schyns et al [2], we show how and when decoding and decorrelation of facial information occur in the brain. For decoding, the left and right hemispheres cooperate to construct contra-lateralized representations of features across spatial frequency bands (e.g. the left eye is represented in the right brain; the right eye in the left brain. Henceforth, we will use a viewer-centered description of features. For example, “the left eye” of a face will be the eye as it appears to the observer.). Irrespective of expression and observer, this construction follows a common routine that is summarized in three stages. Sensitivity to facial features starts at Stage 1 [140–152 ms], which contra-laterally encodes the eyes of the face at a local scale (i.e. high spatial frequencies). Stage 2 [156–176 ms] zooms out from the local eyes to encode more global face information (i.e. using high and low spatial frequencies). Stage 3 [180–200 ms], most critical here, zooms back in to locally and contra-laterally encode the features that individuate each expression (i.e. diagnostic features such as the eyes in “fear”, the corners of the nose in “disgust”, again using higher spatial frequencies). At the end of this time window, the brain has decorrelated the expression signals and has encoded sufficient information to enable correct behavior. In a novel analysis, we demonstrate this point with a Model Categorizer that uses the information encoded in the brain every 4 ms between 140 and 200 ms to attempt to classify the incoming facial expression at 75% correct, as human observers did.

### The face as a transmitter of facial affects

Although humans have acquired the capabilities of spoken language, the role of facial expressions in social interaction remains considerable. For over a century we have deliberated whether facial expressions are universal across cultures, or if expressions evolve within civilizations via biological mechanisms, with Charles Darwin's *The Expression of the Emotions in Man and Animals* [4] central to much of this research. Irrespective of whether facial expressions are inextricably linked to the internal emotion and therefore part of a structured emotional response, or whether cultures develop their own expressions, a facial expression is a visible manifestation, under both automatic and voluntary neural control, that can be measured. The Facial Action Coding System (FACS) details the anatomical basis of facial movement to describe how facial signals are exhibited based on the muscles that produce them. Ekman & Friesen [5] developed FACS by determining how the contraction of each facial muscle transforms the appearance of the face, and how muscles act both singly and in combination to produce cognitive categories of expressions [6].

**Figure 1:**
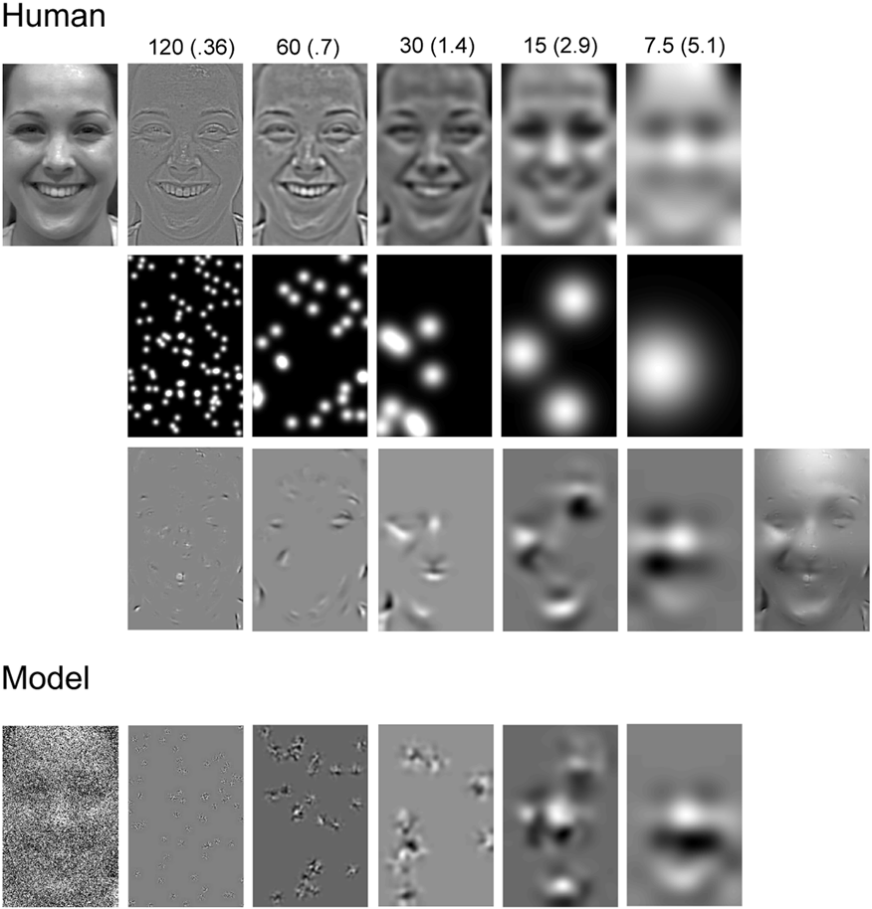
Illustration of Bubbles Sampling [1], [2]. Human. A randomly selected face (from a set of 7 expressions×10 exemplars = 70) is decomposed into six spatial frequency bands of one octave each, starting at 120–60 cycles per face. Only five bands are shown. The sixth band served as constant background. At each spatial frequency band, randomly positioned Gaussian windows (with sigma = .36 to 5.1 cycles/deg of visual angle) sampled information from the face, as shown in the second and third rows of pictures. Summing the pictures on the third row across the five spatial frequency bands plus the constant background from the sixth band produced one experimental stimulus. This is illustrated as the rightmost picture on the third row. Model. The bottom row illustrates the modification of the stimuli to be used in the model. We added white noise to the original picture, which was then decomposed into five spatial frequency bands and sampled with bubbles as described above to produce the experimental stimulus.

**Figure 2:**
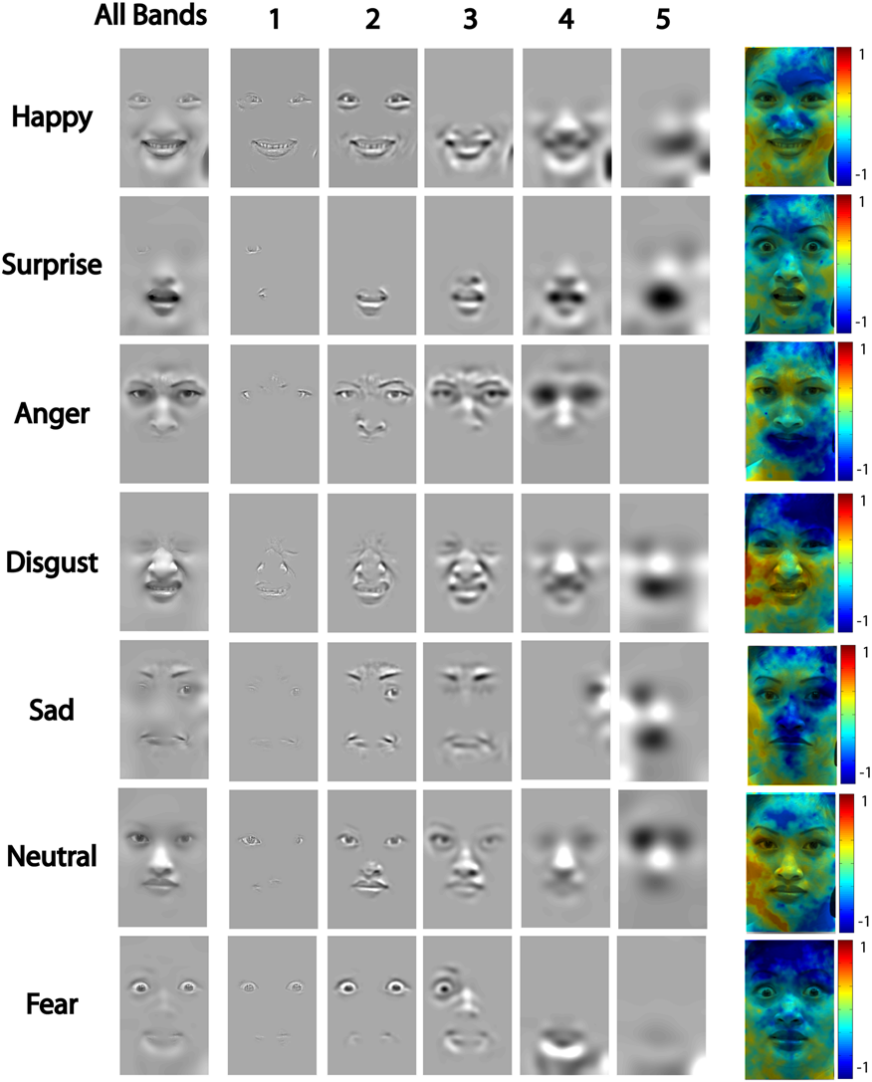
Meta-Analysis of the Behavioral Data. For each spatial frequency band (1 to 5), a classification image reveals the significant (p<.001, corrected, [51]) behavioural information required for 75% correct categorization of each of the seven expressions. All bands. For each expression, a classification image represents the sum of the five classification images derived for each of the five spatial frequency bands. Colored Figures. The colored figures represent the ratio of the human performance over the model performance. For each of the five spatial frequency bands, we computed the logarithm of the ratio of the human classification image divided by the model classification image. We then summed these ratios over the five spatial frequency bands and normalized (across all expressions) the resulting logarithms between −1 and 1. Green corresponds to values close to 0, indicating optimal use of information and optimal adaptation to image statistics. Dark blue corresponds to negative values, which indicate suboptimal use of information by humans (e.g. low use of the forehead in “fear”). Yellow to red regions correspond to positive values, indicating a human bias to use more information from this region of the face than the model observer (e.g. strong use of the corners of the mouth in “happy”).

On this basis, the face can be construed as a signaling system: as a system that transmits a signal about the emotional state of the transmitter. Smith et al [1] measured the characteristics of facial expressions as signals. Using Bubbles ([7] and Figure 1) they sampled information from 5 one-octave Spatial Frequency bands and computed the facial features that observers required to be 75% correct, independently with each of the 7 Ekman & Friesen [3] categories of emotion (see also Schyns et al [2] and Figure 2). In addition, they constructed a model observer that performed the same task, adding white noise to the sampled information to modulate performance. In Smith et al [1] the model observer provides a benchmark of the information that the face signals about each expression. With classification image techniques, Smith et al [1] found that the transmitted information formed a set with low average correlation. That is, they found that different parts of the face transmitted different expressions, resulting in low Pearson correlations between the features of the face transmitting the expressions. For example, the wide-opened eyes were mostly involved in “fear”, the wrinkled corners of the nose in “disgust” and the wide-opened mouth in “happy”. They also found that human categorization behavior depended on using these decorrelated cues.

We have argued that emotion signals have high adaptive value and there is evidence that they have evolved into a lowly correlated set. Turning to the receiver of the emotional signals, i.e. the brain, we can examine the coupling that exists between the encoding of the expression by the face for transmission and the decoding of the signals in the brain. Facial expressions of emotion represent particularly good materials to study the particulars of this transmitter-receiver coupling because most of us are expression experts. Thus, brain circuits are likely to have evolved to decode expression signals fast and efficiently, given the wide range of viewing conditions in which facial expressions are typically experienced. We will briefly review where and when in the brain emotion identification is proposed to happen.

### The brain as a decoder of facial affects: Where are the cortical and subcortical networks?

Emotional stimuli may hold a privileged status in the brain [8], commanding a distributed neural network of cortical and subcortical structures for representing different facial expressions and determining adaptive responses to such stimuli [9], [10], [11], [12] As established by single-cell analysis, neuroimaging and lesion studies, this network has contributions from the amygdala, cingulate gyrus, hippocampus, right inferior parietal cortex, ventromedial occipito-temporal cortex, inferotemporal cortex and the orbitofrontal cortex [12], [13], [14], [15].

With respect to functional roles, the occipito-temporal cortical pathway (in particular the fusiform gyrus and superior temporal sulcus) may be involved in the early perceptual encoding that is essential for differentiating between expressions. Ensuing categorization may require neural structures including the amygdala and orbitofrontal cortex to integrate perceptual encoding of the face with prior knowledge of emotion categories [16], [17], [18].

Another, independent subcortical pathway sidestepping striate cortex could allow a coarse, very fast processing of facial expression. In particular, fear or threat-related signals may benefit from a direct subcortical route to the amygdala, via the superior colliculus and pulvinar thalamus (see [19], [20], [21], [22]). Evidence for a subcortical route arises in part from perception of emotional content without conscious experience, but this is still a controversial topic [21], [22], [23], [24], [25], [26].

### The brain as a decoder of facial affects: When does the decoding happen?

Estimates of the time course of emotion processing in the brain can be derived from event-related potential (ERP) studies. The N170 is a face-sensitive ERP, making it a good candidate to study the time course of emotions signalled by the face. Peaking between 140–200 ms after stimulus onset and occurring at occipito-temporal sites [27], [28], what this potential actually reflects remains somewhat controversial–i.e. if it is linked to the structural encoding of faces, a response to the eyes [27], [29], or whether it can be modulated by emotional facial expression [30], [31], [32]. Despite some studies reporting no effect of emotion, due to its robust sensitivity to faces, the N170 remains a good measure of early facial expression discrimination. Indeed, Schyns et al [2] demonstrated that activity in the 50 ms preceding the N170 peak reflects a process that integrates facial information. This integration starts with the eyes and progresses down on the face. Integration stops, and the N170 peaks, when the diagnostic features of an expression have been integrated (e.g. the eyes in “fear”, the corners of the nose in “disgust” and the mouth in “happy”). Consequently, distance of the diagnostic features from the eyes determines the latency of the N170.

Thus, evidence from brain imaging techniques suggests that cortical and subcortical networks are both involved in the fast processing of emotions. In the cortical route, there is evidence that emotion decoding happens over the time course of the N170 ERP, in a time window spanning 140 to 200 ms following stimulus onset. If the face transmits affects for the visual brain to decode, we must turn to the visual system for an understanding of the specific visual inputs to the network of brain structures involved in this fast decoding.

### Decoding Facial Affects In Visual Signals: The Role of Spatial Frequencies

A classic finding of vision research is that the visual system analyzes the retinal input, therefore including facial expressions, into light-dark transitions, at different spatial frequencies. A set of filters, called “Spatial Frequency Channels”, performs this analysis: Each channel is tuned to a preferential frequency band, with declining sensitivity to increasingly different frequencies. A “bandwidth” characterizes the range of frequencies to which a channel is sensitive, and channel bandwidths are mostly in the range of 1 to 1.5 octaves–where an octave is a doubling of frequency, e.g., from 2 to 4 cycles per deg (c/deg) of visual angle, 4 to 8 c/deg, 16 to 32 c/deg and so forth. In total, approximately six channels constitute the bank of spatial filters analyzing the retinal input (see [33] for a review).

At the centre of the research agenda is the debate of how high-level cognition interacts with inputs from low-level spatial frequency channels to extract information relevant for visual categorization (see [33] for a review). Top-down control implies that the visual system can actively modulate information extraction from one, or a combination of spatial frequency channels for stimulus encoding and categorization. For example, if categorization of “fear” requires extraction of the wide-opened eyes from the retinal input, and because the wide-opened eyes are fine scale features, their accurate encoding should draw information from higher spatial frequency filters. In contrast, the wide-opened mouth of “happy” is a large scale feature allowing encoding to be more distributed across the filters. Top-down control of spatial frequency channels, often cast in terms of modulated attention, implies such flexible tuning of the visual system to encode the combination of spatial channels representing categorization-relevant information (with e.g., involvement of different channels for “the eyes” and “the mouth”).

Several researchers have argued for a special role of the low frequency bands in face processing [34], [35], [36], [37], [38] particularly so in the categorization of facial expressions. Subcortical structures [39], [40], [41], more sensitive to low spatial frequencies [42], [43], would directly activate the amygdala (and related structures [44]) in response to fearful faces represented at low spatial frequencies. Vuilleumier et al [44] also noted a sensitivity of the fusiform cortex to higher spatial frequency ranges, but an implied dissociation of subcortical and cortical pathways to process SF information remains debatable [45]. The idea of a coarse, fast representation via low spatial frequencies [46] finds echo in Bar et al [47] who suggest a fast feedforward pathway to orbitofrontal cortex, which in turn directs precise, high spatial frequency information extraction in the visual input via the fusiform gyrus (see also [42], [48]. So, not only are spatial frequency bands important because they represent the building blocks of visual representations; spatial frequency bands also appear to play a central role in emotion processing in the brain.

### Transmitting Decorrelated Facial Expressions, Constructing their Spatial Frequency Representations in the Brain

We have reviewed the evidence that muscle groups in the face produce lowly correlated signals about the affective state of the transmitter. These facial signals impinge on the retina where banks of spatial filters analyze their light-dark transitions at different scales and orientations. This information is then rapidly processed (i.e. before 200 ms) in cortical and subcortical pathways for the purpose of categorization.

In what follows, we will be concerned with the coupling transmitter-receiver for the categorization of Ekman's six basic expressions of emotion plus neutral. Merging the data of Smith et al [1] and Schyns et al [2], using Bubbles, we will perform an analysis to characterize the facial expressions of emotion across spatial frequency bands as signals. In line with Smith et al [1], we will show that they form a lowly correlated set of signals. We also perform a meta-analysis on the behavioral data of 17 observers to understand how categorization behavior depends on facial cues represented across multiple spatial frequency bands. We will show that their behavior relies on the decorrelations present in the expression signals.

With time resolved EEG, we will then report new analyses in three observers revealing how the left and right occipito-temporal regions of the brain extract facial information across spatial frequency bands, over the first 200 ms of processing, to construct decorrelated representations for classification (see [49] for a generic version of this point). A simple Model Categorizer which uses the information represented in the brain as input will demonstrate when (i.e. the specific time point at which) this representation becomes sufficient for 75% correct categorizations of the seven expressions, as required of the observers' behavior.

## Methods

### Computational meta-analysis: signalling emotions and categorizing them, model and human observers

Using classification image techniques (*Bubbles*, [7]), we will characterize with a model observer the information signalled in Ekman & Friesen [3] six basic categories of facial expressions of emotion plus “neutral”. In addition to characterizing signal transmission, we will characterize, using the same classification image techniques, how behavior uses information from the transmitted signals. These meta-analyses follow the methods developed in Smith et al [1], but pooling data drawn from Smith et al [1] and Schyns et al [2] to provide more power to the analyses.

#### Participants

A total of 7 male and 10 female University of Glasgow students of normal or corrected to normal vision were paid to participate in the experiments. Their behavioural data were pooled from Smith et al [1] and from Schyns et al [2]. For each participant, written informed consent was obtained prior to the experiment and ethics was granted by University of Glasgow FIMS ethics committee.

#### Stimuli

Stimuli were generated from 5 male and 5 female faces, each displaying the six basic FACS-coded [5] emotions plus neutral, for a total of 70 original stimuli (these images are part of the California Facial Expression, CAFÉ, database [50]).

#### Procedure. Human Experiment

On each trial of the experiment (1200 trials per expression in Smith et al [1]; 3000 trials per expression in Schyns et al [2], an original face stimulus was randomly chosen and its information randomly sampled with Bubbles as illustrated in Figure 1. The stimulus was split into five non-overlapping spatial frequency bands of one octave each, starting at 120–60 cycles per image. A sixth spatial frequency band served as constant background. Information was randomly sampled from each band with a number of randomly positioned Gaussian apertures, whose sigma was adjusted across spatial frequency band so as to sample 6 cycles per aperture. The information sampled per band was then recombined to form the stimulus presented on this trial. It is important to note that this version of Bubbles, which samples information across spatial frequency bands, has the advantage of sampling information across the scales of a face. On each trial, global and local face cues are simultaneously presented to the visual system. Thus, both type of cues can by used by face processing mechanisms.

Observers indicated their categorization response by pressing one of 7 possible computer keys. Stimuli remained on the screen until response. A staircase procedure was used to adjust the number of bubbles (i.e. the sampling density) on each trial, so as to maintain categorization performance at 75% correct, independently for each expression. This is important: All observers categorized each one of the seven expressions at the same level of 75% correct performance.

#### Model Observer

Experimental stimulus sets are samples of a population of stimuli. To benchmark the information available in our stimulus set to perform the experiment, we built a model observer. We submitted the model to the same experiment as human observers (collapsed across Smith et al [1] and Schyns et al [2] data), using as parameters the average accuracy per expression (*n* = 17 observers), the average number of bubbles per expression and the total number of trials (25,800) per expression. To maintain performance of the model at 75% correct for each expression, a staircase procedure adjusted a density of white noise, on a trial-per-trial basis, and independently for each expression. On each trial, the model computed the Pearson correlation between the input expression and the 70 original faces revealed with the same bubbles as the input (collapsing all the 380×240 images pixels×5 spatial frequency bands into a 1-dimensional vector). A winner-take-all scheme determined the winning face. Its emotion category was the model's response for this trial. To the extent that the model compares the input to a memory of all stored faces, it can use all the information present in the original data set to respond. Thus, the model provides a benchmark of the information present in the stimulus set to perform the expression categorization task.

### Computational analysis: time course of spatial frequency processing in the brain to encode emotion signals

Having shown that behavioural categorization performance requires lowly correlated emotion signals to be correct, we now turn to brain data (i.e. the EEG of three observers from Schyns et al [2] to understand how this decorrelation is accomplished.

#### Participants

The three participants from Schyns et al [2], whose data served in the behavioural meta-analysis, had their scalp electric activity recorded on each trial of the expression categorization task described before (with 3000 trials per expression).

#### EEG Recording

We used sintered Ag/AgCl electrodes mounted in a 62-electrode cap (Easy-Cap™) at scalp positions including the standard 10-20 system positions along with intermediate positions and an additional row of low occipital electrodes. Linked mastoids served as initial common reference, and electrode AFz as the ground. Vertical electro-oculogram (vEOG) was bipolarly registered above and below the dominant eye and the horizontal electro-oculogram (hEOG) at the outer canthi of both eyes. Electrode impedance was maintained below 10 kΩ throughout recording. Electrical activity was continuously sampled at 1024 Hz. Analysis epochs were generated off-line, beginning 500 ms prior to stimulus onset and lasting for 1500 ms in total. We rejected EEG and EOG artefacts using a [−30 mV; +30 mV] deviation threshold over 200 ms intervals on all electrodes. The EOG rejection procedure rejected rotations of the eyeball from 0.9 deg inward to 1.5 deg downward of visual angle–the stimulus spanned 5.36 deg×3.7 deg of visual angle on the screen. Artifact-free trials were sorted using EEProbe (ANT) software, narrow-band notch filtered at 49–51 Hz and re-referenced to average reference.

#### Computation: Sensor-based EEG Classification Images

To determine the facial features systematically correlated with modulations of the EEG signals, we applied Bubbles to single trial raw electrode amplitudes. We selected, for each observer, a Left and a Right Occipito-temporal electrode (henceforth, OTL and OTR) for their highest N170 amplitude peak on the left and right hemispheres (corresponding to P8 and PO7 for each observer).

For each electrode of interest, EEG was measured every 4 ms, from −500 ms to 1 s around stimulus onset. For each time point, and for each expression and SF band, we computed a classification image to estimate the facial features correlated with modulations of EEG amplitudes. This classification image was computed by summing all the bubble masks leading to amplitudes above (vs. below) the mean, at this time point. We repeated the procedure for each one of the five spatial frequency bands and for each one of the seven expressions and each one of the 250 time points. Subtracting the bubbles masks above and below the mean leads to one classification image per SF band, time point and expression. This classification image represents the significant (p<.05, corrected, [51]) facial information (if any) that is correlated with modulations of the EEG for that SF band, time point and expression (see Schyns et al [2], [53]; Smith et al [1], for further details).

## Results

### Computational meta-analysis: signalling emotions and categorizing them, model and human observers

We performed the same analysis for the human and model observers. To illustrate, consider the analysis of the expression “happy” in the top row of Figure 2. In each spatial frequency band, and for each pixel, we compute a proportion: the number of times this pixel led to a correct response over the number of times this pixel has been presented. Remember that performance was calibrated throughout the experiment at 75% correct. On this basis, we determine the informative from the noninformative pixels of an expression: the proportion associated with informative pixels will be above .75. In each Spatial Frequency band, we found the statistically relevant pixels (corrected, [51], *p*<.001). The first row of images (labelled 1–5) illustrates these pixels on one exemplar of the “happy” category, for each Spatial Frequency band. The sum of these images (under “all bands”) summarizes the information that the observers required for 75% correct categorization behavior. To illustrate, “happy” requires the smiling mouth and the eyes, “surprised” the open mouth, “anger” the frowned eyes and the corners of the nose, “disgust” the wrinkles around the nose and the mouth, “sadness” the eyebrows and the corners of the mouth and “fearful” the wide-opened eyes.

These facial features, in the context of equal categorization performance across expressions, provide the information that human observers required to choose amongst seven possible expressions, without much expectation as to what the input expression would be. A remaining question is the extent to which other information exists, between the seven emotion categories of this experiment, to accomplish the task at the same performance level (here, 75% correct). To this end, we turn to the model observer for which we performed an identical analysis as that described for human observers, searching for pixels at the same level of statistical significance (corrected, [51], *p*<.001). For each expression (rows in Figure 2) and Spatial Frequency band (columns of Figure 2) we computed a measure of the optimality of information use by human observers: the logarithm of the ratio between human significant pixels and model significant pixels. Adding these logarithms across spatial frequency bands per expression and applying them to an original stimulus reveals in green an optimal use in humans, in blue, a suboptimal use of information by humans (e.g. the corners of the nose in “happy”, the mouth region in “anger”, the frown of the forehead in “fear”) and in red a particular bias to use information from this region, at least more so than the model observer (e.g. the corners of the mouth in “happy” the wrinkle of the nose and the mouth in “disgust”). Such human biases might reveal that in a larger population of stimuli, this information would be more informative than in the stimulus set of this particular experiment.

Having characterized the use of facial information for expression categorization, we turn to the effectiveness of the face as a transmitter of emotion signals and introduce a new measurement for the effectiveness of the signals themselves. In signal processing, the more dissimilar the encoding of two signals the less confusable they are in transmission. An ideal set of signals is “orthogonal” in the sense that they are fully decorrelated. To evaluate the decorrelation of the six basic expressions of emotion plus neutral, we examined the decorrelation of the significant pixels of the model observer. To this end, we turn the 380×240 image pixels×5 Spatial Frequency bands representing each expression into a one-dimensional vector of 38×24×5 entries (by compressing the images by a factor of 10), and cross correlate (Pearson) them to produce a symmetric correlation matrix where each (x, y) correlation represents the similarity between two expressions. If the expressions formed an orthogonal set, then the correlation matrix should be the identity matrix, with correlations (x, y) = 0 for each pair of expression, and (x, x) = 1 on the diagonal, for the correlation of each expression with itself. The Backus-Gilbert Spread [52] measures the distance between the identity matrix and an observed matrix. We adapted a Backus-Gilbert Spread to provide a measure of decorrelation (1).
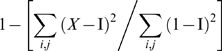
(1)


**Figure 3:**
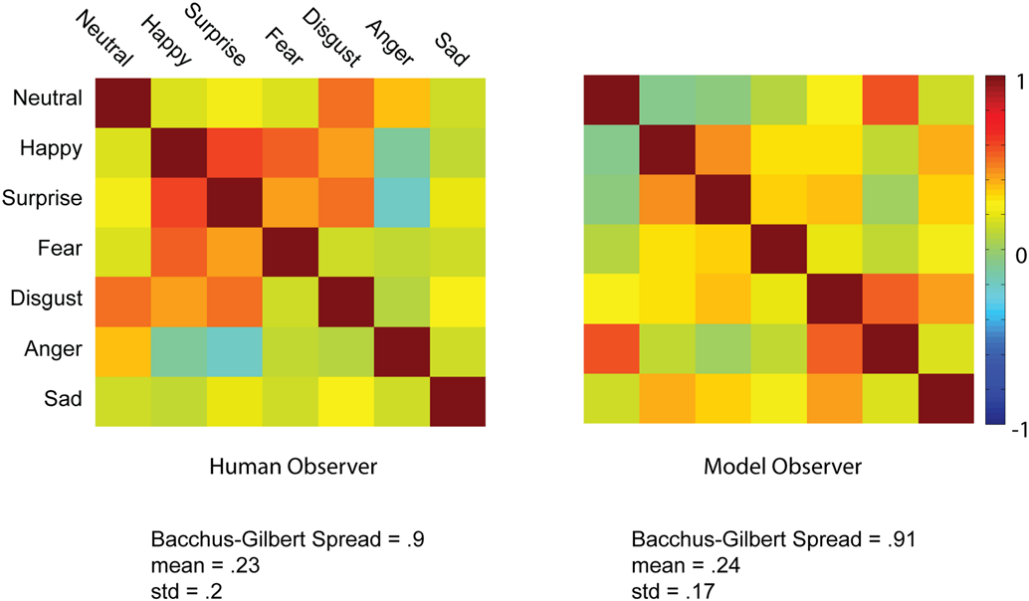
Meta-Analysis of the Behavioral Data: Cross-Correlations of the Classification Images. For both the human and the model observers, we transformed the 380×240 pixels×5 spatial frequency bands classification images into a one-dimensional 38×24×5 vector. We Pearson-correlated these vectors to produce the correlation matrices shown in the figure, with correlation values ranging between 1 (in dark red) and −1 (in dark blue). Backus-Gilbert Spread of the correlation matrix is reported together with mean correlation and standard deviation.

Figure 3 illustrates the correlation matrices and their respective Backus-Gilbert Spread measurement of decorrelation (maximum decorrelation is 1; minimum decorrelation is 0). For human and model observers, Backus-Gilbert Spread was high, indicating high decorrelation of the facial features represented across spatial frequencies for the different expressions. The mean pairwise correlation was therefore low for both the human (m = .23; std = .2) and model (m = .24; std = .17) observers. So, it is clear that humans are particularly adapted to the task of extracting the information that is available in the stimulus set of facial expressions to decorrelate these categories. It is important to note that our correlations are based on the locations of the diagnostic pixels for the expressions, not on the expressions themselves. Our correlations therefore probably overestimate the correlations between the expressions. To illustrate, although both “fearful” and “anger” use information from the eye region and so would be correlated, the wide opened eye and the eye with eyebrows are quite different.

From the model observer, we conclude that on the transmitting end the brain has evolved to transmit basic expressions of emotion that are lowly correlated. On the receiving end, the behavioural data of human observers reveal that the brain has evolved routines to decorrelate facial emotion signals for adapted categorization behavior.

### Computational analysis: time course of spatial frequency processing in the brain to encode emotion signals

#### a. Spatial Frequency Use In the Brain

**Figure 4:**
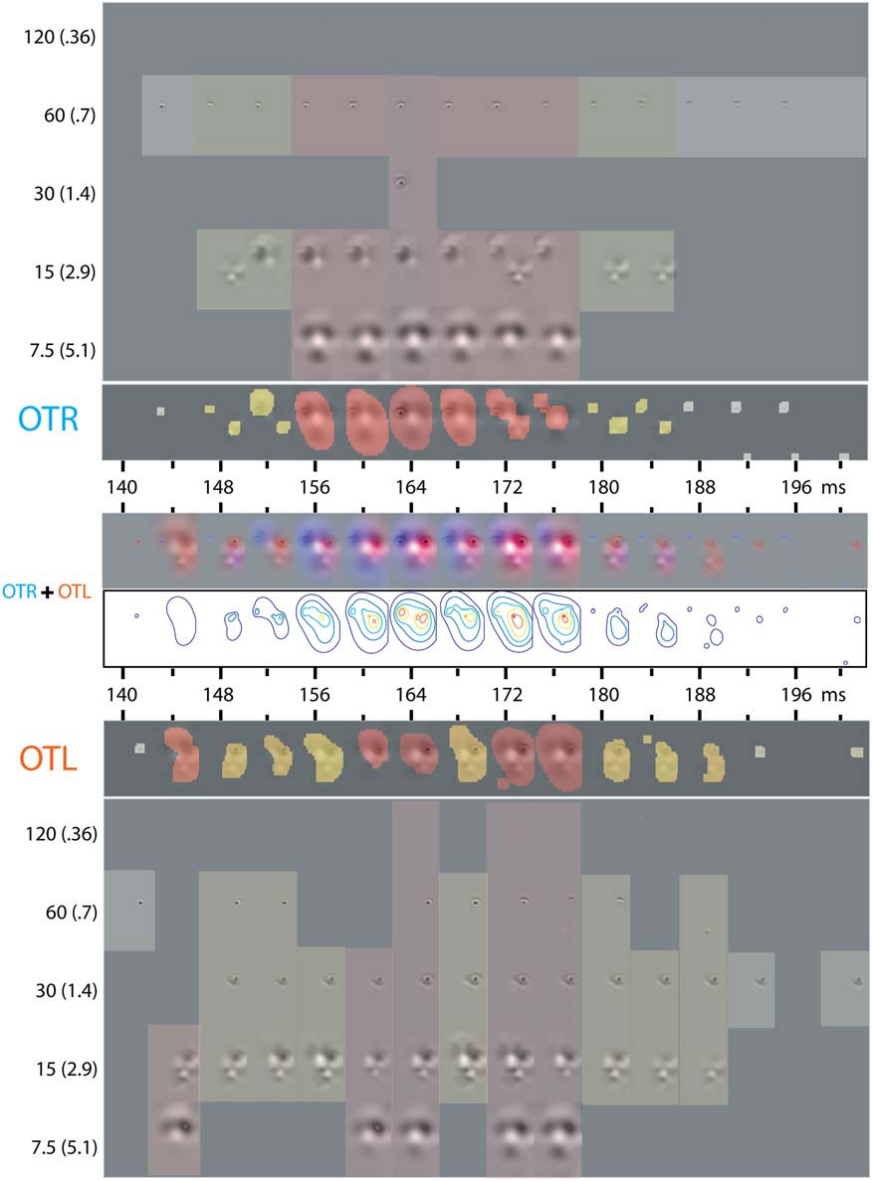
Analysis of the EEG Data: From EEG Classification Image to Combinations of Spatial Frequency Bands. On each of 3000 trials per expression, facial information was sampled from one of 70 original stimuli (5 males and 5 females, each displaying one of 7 expressions) using Gaussian windows from 5 non-overlapping Spatial Frequency bands of one octave each, starting from [120 to 60] cycles per face—i.e., [.36 to .7] degrees of visual angle. For each SF band and every 4 ms, we computed an EEG classification image, thresholded at *p*<.05 (illustrated here for PO7, Occipito-temporal Left, OTL and P8, Occipito-temporal Right, OTR electrodes), for each expression (illustrated for “fear”). OTR; OTL. We added the classification images in each column, across spatial frequency bands, to produce the Occipito-temporal Left and Right classification image movies. The white-to-red color code illustrates the particular combination of spatial frequency band(s) to which the EEG is sensitive at each time point. Note that the OTR (vs. OTL) electrode is contra-laterally sensitive to the left (vs. right) eye. OTR+OTL. To depict the respective contributions of the left and right hemispheres to the encoding of expression features (in the illustration, the left and right eyes), at each time point we added the OTR and OTL classification images and color-coded them (OTR contribution in blue; OTL contribution in red). Contour plots depict the local and/or global spatial extent of the encoding process. We repeated this analysis independently, on 64 electrodes, for each one of 3 observer and 7 expressions.

**Figure 5 pone-0005625-g005:**
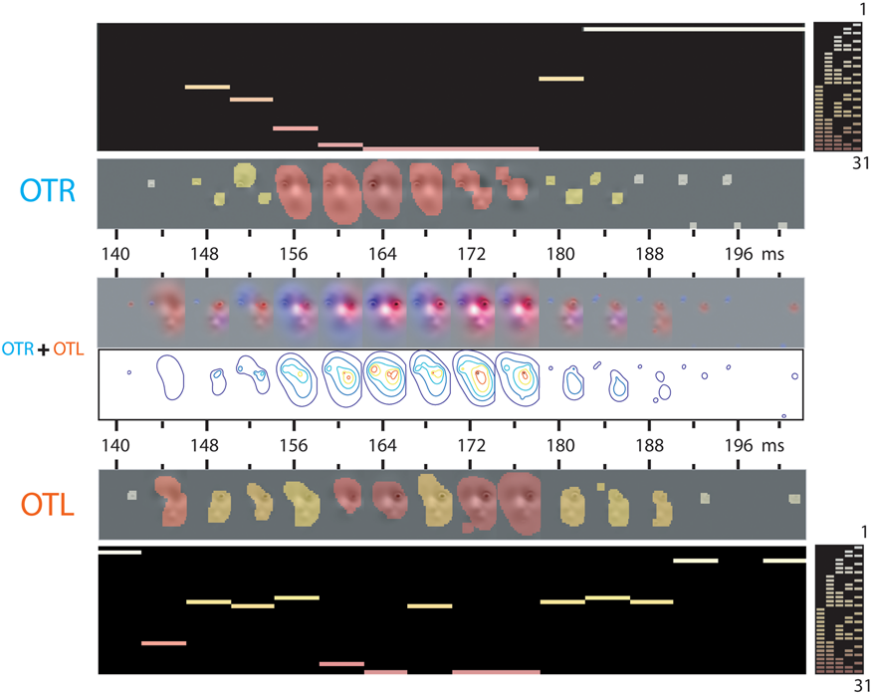
Analysis of the EEG Data: Time Course of the Sensitivity to Combinations of Spatial Frequency Bands (Observer LP, “fear”). Every 4 ms, on electrode OTL and OTR, we represent the combination of the five spatial frequency bands with a binary number (in decimal between 1 and 31) and color code it between white (1) and red (31), see Figure 4 for details. OTR+OTL. To depict the respective contributions of the left and right hemispheres to the encoding of expression features (in the illustration, the left and right eyes), at each time point we added the OTR and OTL classification images and color-coded them (OTR contribution in blue; OTL contribution in red). Contour plots depict the local and/or global spatial extent of the encoding process.

We aim to understand how the decoding routines of the brain decorrelate facial expressions of emotion, using spatial frequency bands, the early building blocks of visual processing. Figures 4 and 5 illustrate the analysis for the N170 time window of interest (see [54] for the full time course on all electrodes). For each observer and expression (here, illustrated with LP and “fearful”), on electrodes OTR and OTL, a classification image is computed every 4 ms of the N170 time course to represent the sensitivity of the EEG across the five Spatial Frequency bands of the input. We represent the specific combination of Spatial Frequency bands composing the OTR and OTL classification images at each time point with a binary code (with decimal values comprised between 1 and 31). To illustrate, on OTL, at 140 ms, the EEG is sensitive to the contra-lateral right eye (local information) at Spatial Frequency band 2. We represent this with binary code 00010 (2 in decimal) and color-code it in pale yellow. At 172 ms, still on OTL, the EEG is sensitive to the contra-lateral right eye and neighbouring information (more global information) *across all SF bands*, represented with binary code 11111 (31 in decimal) and color-coded in red. At 192 ms, sensitivity reverts back to the local processing of the contra-lateral eye, at a medium frequency band represented with binary code 00100 (8 in decimal). Note on OTR the complementary, contra-lateral encoding of the left eye, following the same high spatial frequency (local processing), then most frequency bands (local and global processing) then high spatial frequency again (local processing). In addition, to depict the respective contributions of the left and right hemispheres to the encoding of expression features, at each time point we added the OTR and OTL classification images and color-coded them (OTR contribution in blue; OTL contribution in red). Contour plots depict the local and/or global spatial extent of the encoding process in the face.

**Figure 6 pone-0005625-g006:**
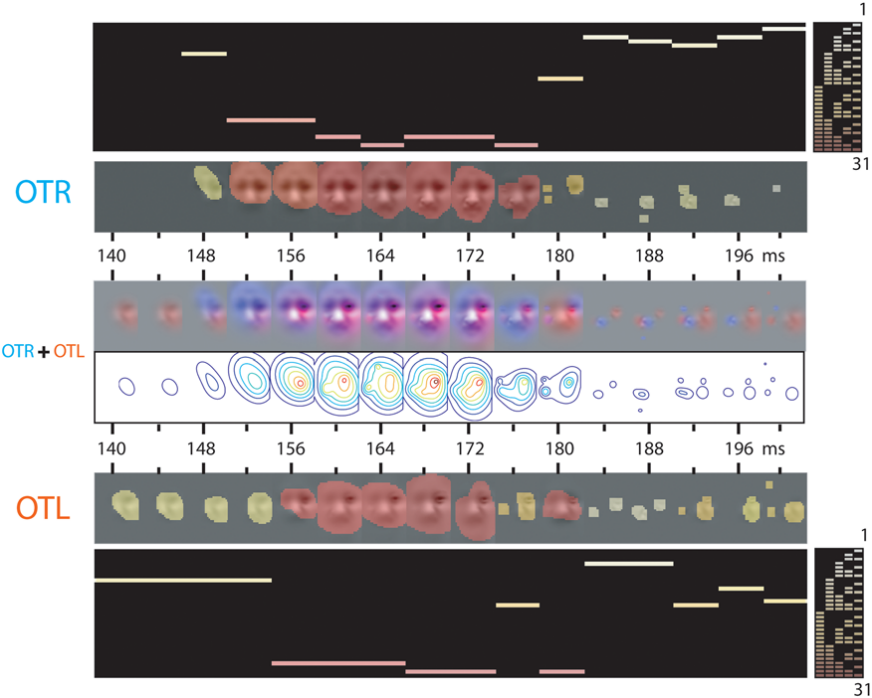
Analysis of the EEG Data: Time Course of the Sensitivity to Combinations of Spatial Frequency Bands (Observer LP, “disgust”).

**Figure 7:**
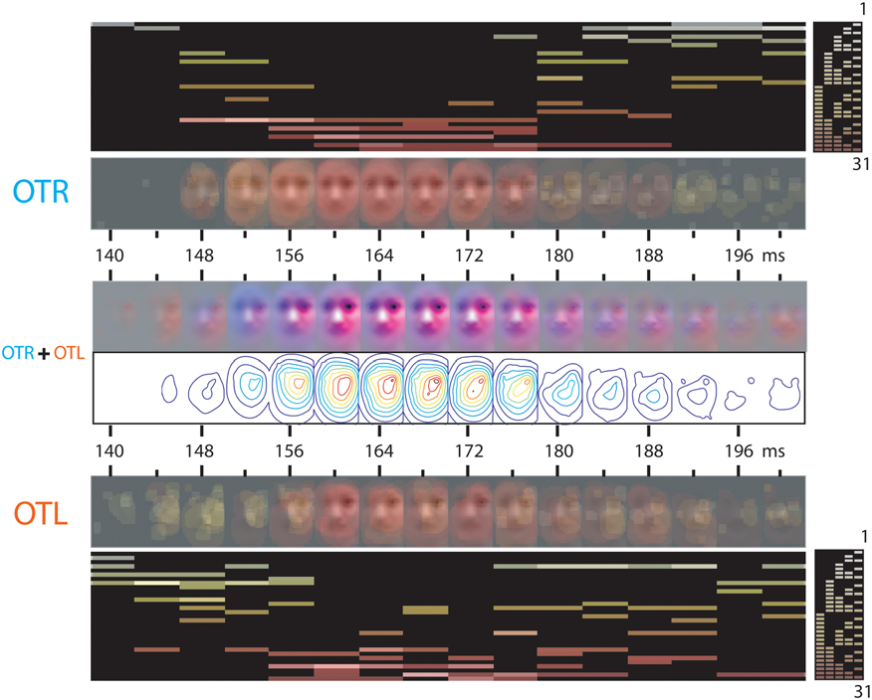
Analysis of the EEG Data: Time Course of the Sensitivity to Combinations of Spatial Frequency Bands (Observer LP, collapsed across all seven expressions). For each electrode, we collapsed the time course of sensitivity to the combinations of spatial frequency bands across the seven expressions (see Figure 5 and 6 for individual examples). OTR+OTL. To depict the respective contributions of the left and right hemispheres to the encoding of expression features, at each time point we added the OTR and OTL classification images across the seven expressions and color-coded them (OTR contribution in blue; OTL contribution in red). Contour plots depict the local and/or global spatial extent of the encoding process.

Figure 5 summarizes the analysis just described on Figure 4, with the combination of SF bands represented at each time point with a color-coded bar in binary coding space. Figures 5 and 6 illustrate the analysis, for observer LP and expressions “fear” and “disgust” (Figures S1, S2, S3 and S4 illustrate the equivalent data for observers LF and UM). The binary coding is a useful space to summarize the time course of the sensitivity of the N170 to specific combinations of spatial frequency bands. The pattern noted above of high spatial frequency to full spectrum and back to high spatial frequency (or local to local_and_global and back to local processing) characterizes processing in the left and right occipital regions of all three observers, for “fear” and “disgust” and in fact, for all seven expressions. Figure 7 presents for observer LP the binary code data collapsed across all seven expressions (grey-levels of the binary coding represents frequency of each code, see Figures S5 and S6 for observers LF and UM).

Together, the results presented in figures 4 to [Fig pone-0005625-g005] [Fig pone-0005625-g006] 7 (and corresponding Figures S1, S2, S3, S4, S5 to S6) reveal a similar pattern of sensitivity to spatial frequency combinations on the left and right hemispheres: Starting with a combination of few high spatial frequency bands, face encoding starts at a local scale with the eyes, around 140 ms following stimulus onset. Around 156 ms, encoding zooms out from the local eyes to encode more global information, using all (or most) spatial frequency bands. Around 180 ms, encoding zooms back in, at a local scale, with sensitivity to few high spatial frequency bands. For all three observers, all seven expressions, the pattern of spatial frequency sensitivity, over the N170 time course, is local (few high spatial frequency bands) to local-and-global (all spatial frequency bands) to local again (few high spatial frequency bands).

#### b. Decorrelation of Expressions Cues from Spatial Frequency Information

The sensitivity to combinations of spatial frequency bands just revealed reflects a mechanism that processes information over the face. Our goal now is to assess whether this brain mechanism seeks to decorrelate internal representations of facial expressions. A high decorrelation would imply that the brain has encoded the information required to individuate and behaviorally categorize all seven expressions.

**Figure 8:**
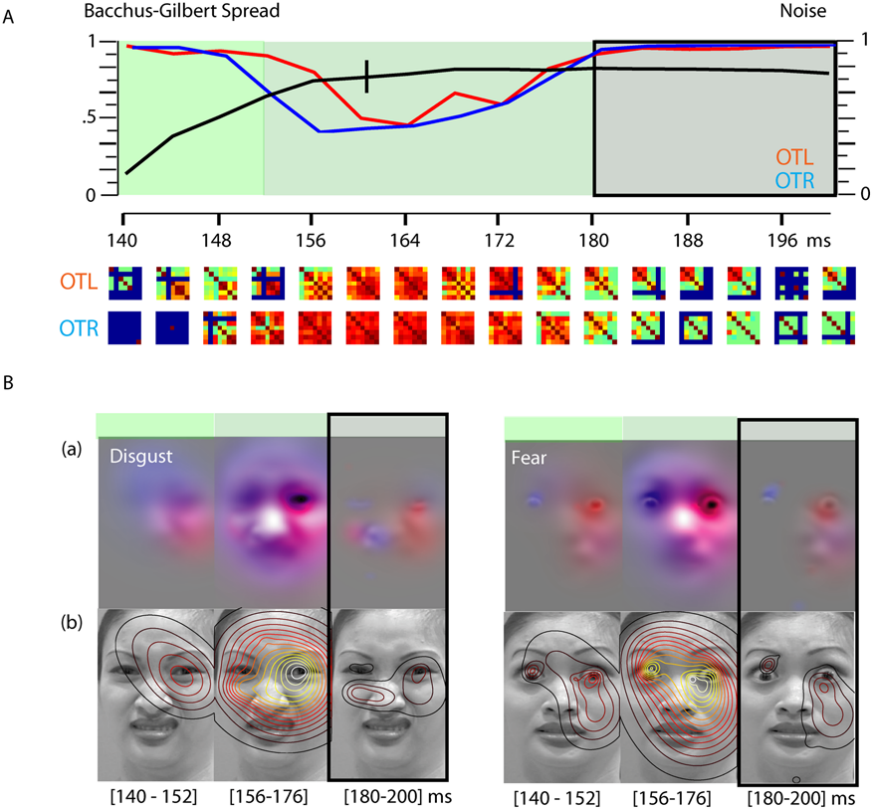
Meta-Analysis of the EEG Data: Decorrelation of Facial Expressions Between 140 and 200 ms Following Stimulus Onset. A. Backus-Spread Measure of Decorrelation of Facial Expressions (Observer LP). At each time point, for each expression, we transform the classification image (OTR+OTL) into a single high-dimensional vector (of 38×24 image pixels×5 spatial scales of dimensionality). We then cross-correlate the vector for each expression to generate a 7×7 cross-correlation matrix (displayed in the Figure for each time point and electrode OTR, OTL). If the brain aims to individuate expressions, it should decorrelate its representations. In computational terms, the cross-correlation matrices should evolve towards the identity matrix over time (with correlation = 0, in blue, for each pair of expressions; correlation = 1, in red, for self-correlations). Backus-Gilbert Spread (6) measures this distance between the identity and observed matrices. Between 140 and 200 ms following stimulus presentation, the measure identifies three time intervals of decorrelation, represented in a “V”-shaped curve on OTL (red curve) and OTR (blue curve). The black curve reveals the performance of the Model Categorizer which predicts the emotion category from the OTL+OTR classification images plus noise—i.e. from the representation of the expression constructed in the brain. The categorizer was a Winner-Take-All scheme which compared (Pearson correlated) the noisy input with the 70 original stimuli and adjusted noise level, independently for each expression and time point, to maintain classification at 75% correct. The increase of noise level with time (averaged across all 7 expressions) reveals that the representations constructed in the brain are sufficient for behavior and become more robust with time. The vertical marker on the black curve indicates the time point from which 75% correct performance can be achieved. B. Three Stage of Spatial Frequency Sensivitity in the EEG. The “V” shaped Backus Gilbert curves identify three time intervals of decorrelation (color-coded in green). (a) Within each time interval, we averaged the classification images and color-coded them for the respective contribution of OTR (blue) and OTL (red). These illustrate the time course of the decorrelation process for the facial features of “disgust” and “fear”. (b) The contour plots reveal, in both expressions, that Stage 1 [140–152 ms] contra-laterally encodes the eyes of the face at a local scale. Stage 2 [156–176 ms] zooms out from the local eyes to encode more global face information. Stage 3 [180–200 ms], highlighted with a black box, zooms in to locally and contra-laterally encode the features individuating each expression with maximum decorrelation—i.e. the eyes in “fear” and the corners of the nose in “disgust”.

To this end, for each observer, every 4 ms, independently for electrode OTR and OTL, we transformed the 380×240 pixels×5 spatial frequency bands representing each expression into a one-dimensional vector with 38×24×5 entries, and cross correlate (Pearson) them to produce a symmetric correlation matrix per electrode where each (x, y) correlation represents the similarity between two expressions. For each observer, we applied the Backus Gilbert Spread measure as explained for behavior, independently on OTR and OTL, every 4 ms between 140 to 200 ms, to derive a curve of decorrelation (1). Figure 8 presents the data of observer LP (and Figures S7 and S8 for observers LF and UM).

The Backus-Gilbert Spread plots in Figure 8 and Figures S7 and S8 reveal similar V-shaped dynamic patterns. These dynamics imply an initial stage of decorrelation, following by a stage of strong correlation, ending with a stage of decorrelation. To facilitate interpretation and reduce the complexity of the analysis, we segment the time course of the Backus-Gilbert Spread into three distinct periods, roughly corresponding to the two inflexion points of the curves: Stage 1 [140–152 ms], Stage 2 [156–172 ms], Stage 3 [180–200 ms]. Within these time intervals, we average for each observer the OTR and OTL classification images of illustrative expressions (“fear” and “disgust”) and color-code them. Finally, we display the contour plots of scale processing associated with these illustrative examples to complete the interpretation.

The OTR (blue) and OTL (red) Backus-Gilbert Spread curves reveal that the process of decorrelating facial expressions happens in parallel on the left and right occipito-temporal electrodes PO7 and P8, over a 50 ms time window spanning 140 to 200 ms following stimulus presentation, in a process shared between the left and right hemispheres. Specifically, the left and right hemispheres cooperate to construct contra-lateralized representations of information (e.g. the left eye is represented in the right brain; the right eye in the left brain, see Figure 8). Irrespective of expression and observer, this construction follows a common routine that is summarized in three stages. Sensitivity to facial features starts at Stage 1 [140–152 ms], which contra-laterally encodes the eyes of the face at a local scale. Stage 2 [156–176 ms] zooms out from the local eyes to encode more global face information. Stage 3 [180–200 ms], most critical here and highlighted by a black box, zooms back in to locally and contra-laterally encode the features that individuate each expression (i.e. diagnostic features such as the eyes in “fear”, the corners of the nose in “disgust”, see Figure 1).

#### c. Robustness of Early Brain Representations and Usefulness for Behavior

We have shown in the previous section that the left and right hemispheres progressively construct, over 16 time points of 4 ms between 140 and 200 ms following stimulus onset, decorrelated representations of the seven expression categories. We now examine how the information accrued in the brain at each time point (summing the OTL and OTR classification images) can predict the observer's categorization behavior—i.e. 75% categorization accuracy for each expression. We also examine how robust the representations are to noise. Robustness to noise informs on the *efficiency* of a representation for a given task: More efficient representations will typically tolerate more noise for a given level of performance (here, 75% accuracy).

To predict categorization behavior from EEG classification image data, we developed a Winner-take-all Model Categorizer similar to that described in the section *Model Observer* presented earlier (see Figure 1). The difference was that the model's inputs were not the original face pictures plus noise, but the sum of the OTL and OTR classification images (derived for this observer, for this expression and at this particular time point, see OTL+OTR on figures 4 to [Fig pone-0005625-g005] 6, for examples) to which we added white noise using a stair-case procedure (see *Model Observer* earlier). Repeating the simulation independently for each observer and time point, we (1) determine when, between 140 and 200 ms, brain representations can predict the observer's 75% correct behavior and (2) how much additive noise is then required to maintain the model's performance at 75% with each expression, expecting an increase in noise if the representations become more efficient.

We ran a total of 600 trials for each combination of observer, expression and time point. We then averaged, for each observer and across all seven expressions, the added level of noise (represented as a value of sigma between 0 and 1) required to maintain 75% correct categorization at each of the 16 time points. Results appear as black curves in Panel A of Figure 8 (see also Figures S7 and S8). From the time point when 75% correct performance was achieved (marked as a vertical bar on the black curve), added noise will tend to slowly increase over time to maintain constant performance, reflecting progressively more efficient internal representations. See the “noise” curve in Figure 8 (see also Figures S7 and S8).

In sum, analyses of the EEG signal of observers categorizing facial expression have revealed a process whereby (1) the brain progressively decorrelates representations for behavior between 140 and 200 ms following stimulus onset; (2) these representations follow a common routine of spatial frequency extraction (starting with local to the eyes, then local and global to the face and then local again to the diagnostic features); (3) these representations become sufficient to warrant behavioural performance and progressively more robust to additive noise.

## Discussion

If the face evolved in part to transmit the relevant internal emotional states of primates, then their brains probably co-evolved as fast and efficient decoders of facial expressions. We tested these claims using models and the behavioural and time-resolved EEG data of human observers. A model observer demonstrated that the face, as an organ of emotion transmission, sends a different signal for each of the basic expressions of emotion. The model demonstrated that the diagnostic signal was located in different regions of the face for each emotion category, implying that evolution engineered the face to transmit expression signals as a lowly correlated set. To understand how the brain decodes the signals, we first meta-analyzed the behavioural data of 17 observers confronted to the 7 facial expression categories. We showed that their correct behavior depended on the proper extraction of the diagnostic, decorrelated information from the faces. Turning to the brain to finely understand the time course of the information extraction mechanisms, we examined the first signal known to be robustly sensitive to visual event categories, the N170 [55]. We found, in the left and right occipito-temporal regions, that the decoding process functioned as a decorrelation over three main time stages. The first stage, starting about 140 ms following stimulus onset, represents the eyes, irrespective of expression, using combinations of high-spatial frequency information (a local process). Around 156 ms a second stage zooms out from the local eyes to represent more global information using most spatial frequency bands. Around 180ms, encoding zooms back in, at a local scale to represent diagnostic features. We further showed that at this time point, for each observer, the representations of the 7 expressions were maximally decorrelated across both hemispheres. A model categorizer further demonstrated that the brain representations predicted 75% correct behavioural performance and were progressively more robust to added noise. To conclude, the face transmits lowly correlated signals of internal emotional states. The brain of the receiver decorrelates these signals, early on, to categorize them.

### a. Attention to Features, Spatial Frequency Processing, Global-to-Local

As discussed there is a debate in the literature over whether attention to spatial frequency information in the brain is under top-down cognitive control. Intertwined with this debate is the open question of precisely what is being attended: most studies implicitly assumed that it is a combination of spatial frequency bands (at least this is what their design can demonstrate when they test recognition against low vs. high spatial frequency versions of the original stimuli). But we have proposed that what could be attended are specific features (e.g. the eyes or the mouth) represented across several spatial frequency bands [1], [2], [7], [33], [56]; see Ullman et al [57] for computational evidence that such visual features of intermediate complexity are successful in visual classification).

The behavioural data and the model observer presented here offer an unequivocal answer: Attention to spatial frequency information is under top-down cognitive control to extract the combination of spatial frequency information identifying each expression. The object of attention is a number of facial features (e.g. the wide opened eyes in “fear”, the corners of the nose in “disgust” and the smiling mouth in “happy”) that are themselves represented across a number of spatial frequency bands. Turning to brain data, we find further evidence for top-down guidance of attention to features, because the process of representing expressions in the brain finishes with the encoding of the diagnostic, expression specific information, independently in the left and right hemispheres. And these diagnostic features are represented across several spatial frequency bands. However, the process leading to this end point appears to be much more automatic in nature. Irrespective of expression, it starts locally with the eyes, using combinations of high spatial frequencies (or at least few frequencies) in a first stage. In the second stage, again irrespective of expressions, encoding uses most spatial frequency bands to zoom out from the eyes to the face, again independently in the left and right hemispheres. This more automatic process, never demonstrated before is akin to the zoom-lens metaphor of attention with one caveat: When it zooms out from the eyes to the entire face, it still keeps a high resolution on the eyes, whereas the background face is in low resolution. This suggests an increase of the span of attention to locate the diagnostic features, to which the third stage zooms back in, at a high local resolution.

Morrison and Schyns [58], following Schyns & Oliva [46]; Oliva & Schyns [59]) discussed two possible accounts of spatial frequency use for visual categorization: fixed (coarse-to-fine) or flexible, determined by diagnostic information. Coarse-to-fine is the “fleshing of the skeleton” metaphor in which the initially encoded low spatial frequencies of the stimulus are later fleshed out by high spatial frequency details (see also [47]). Flexible is the top-down, cognitively controlled extraction of whichever spatial information suits the needs of the observer in the task considered. Our EEG data depict a more complex picture of spatial frequency use in the brain. As discussed, we find flexibility in Stage 3, when different diagnostic cues are flexibly encoded for each expression. But we find a fixed pattern of spatial frequency use over Stages 1 to 3, which is the same for each expression and observer, independently in their left and right hemispheres. This pattern is not a fixed coarse to fine and it is not fine to coarse. At least in the case of faces, it is a more complicated interaction between what appears to be the requirements of locating automatically landmark facial features such as the eyes (which are fine scale features) to expand out from these to category specific diagnostic features. Future research should attempt to understand the origin of control, both for the apparently automatic and the more strategic attention to features and the dynamics of this process over the spatial frequency space.

It must be noted that the frequency bands chosen to decompose the face stimuli with Bubbles ascribe a relative “high” to “low” spatial frequency content to stimulus information (see Figure 1). This spatial frequency content is a function of the angular dimension of the stimulus as it projects on the retina of the observer. We have recently demonstrated that the categorization accuracy of the seven facial expressions was dependent on viewing distances [60]. This implies that diagnostic features have a particular scale making the recognition of certain expressions more proximal (e.g. “sad”, and “fear”) than other expressions (e.g. “happy” and “surprise”) that can be recognized over a range of viewing distances. It therefore remains an open question to understand how a change in the size of the expressive faces (e.g. dividing or multiplying their size by a factor of two) will change the pattern of spatial frequency processing reported here for each expression (because changing stimulus size changes the spatial frequency composition of diagnostic features projecting on the retina).

### b. Processing of Features; Time Course of Expression Categorization

The data demonstrate that the categorization information is available, but split across the two hemispheres around 200 ms following stimulus onset. We have shown that the brain has in principle accrued sufficient information to perform robust categorizations. This raises a number of questions about the relationship between this information and behavior.

A first question is whether “fear”, assumed to be sub-cortically processed faster than other expressions, could also be processed faster in our cortical model? An argument could be put that the diagnostic features of “fear” are the wide-opened eyes. Given that the eyes are the landmark features that are first encoded for all expressions in Stage 1, there could be a considerable (around 50 ms) processing gain for this expression. However, behavioral data did not reveal significant reaction time differences among expressions. EEG classification images reveal that despite initial encoding of the eyes in Stage 1, encoding zooms back to the eyes at Stage 3. Analyses of the ERP latencies in Schyns et al [2] showed that “fear” elicited some of the fastest ERP peaks, but these peaks all happened at Stage 3 and the differences between slowest to fastest peak were of the order of 20 ms. Our data do not provide evidence for a faster cortical route for “fear” and of course they do not inform the subcortical hypothesis.

A second question is whether information represented across the hemispheres must first be integrated in the brain before categorization decision is made. This is an interesting problem with many ramifications, including conscious perceptions [61], [62]. Our intention here was to examine whether and when the brain orthogonalizes it's decoding of expression signals for the purpose of categorization, not examine when categorization happens. However, using a paradigm similar to Gerson et al [63] we could reverse the analysis in time, using reaction time as a “trigger” (not stimulus onset). We would search for a specific time window over all electrodes, going backwards in time from response to the N170 time window examined here. In this critical time window, we should find evidence for an integration of the information from the OTL and OTR electrodes reported here. This window, assumed to happen shortly after the N170 time window, would provide the first evidence of bi-lateral, integrated diagnostic information. If the time window co-varied with reaction time, then we would have a handle on when integration for categorization behavior is performed.

### c. Categorization Tasks, Available Facial Information and Diagnostic Information

Strictly speaking, the reported data are only valid under the specific conditions of experimentation tested here. As discussed in Schyns' ([64]; see also Gosselin & Schyns [65]) Diagnostic Recognition framework, any categorization task involves three components of visual information. The first component is the information available in the stimulus set to resolve the task (and this information can be severely restricted if the sample of stimuli is not representative of its population). Here, the model observer revealed what this information could be for each expression (see Figure 2). The second component is the information that the observer expects in the input to categorize the stimulus, in the context of the other categories to discriminate in the task (e.g. the wide-opened eyes in “fear” vs. the wide-opened eyes and the open mouth in “surprise”). The third component is the subset of the available information that the observer uses for categorization: the diagnostic information as revealed from the behavioural analysis of bubbles data. It should be clear that changing the categorization task (e.g. discriminating “fear” from “happy” vs. discriminating “fear” from “surprise”) changes the information that is diagnostic. For example, the wide-opened eyes are sufficient for “fear” when discriminating it from “happy” but the eyes and the mouth might be checked from the same fearful face, if it is to be discriminated from “surprise” (the eyes display highly correlated information between “fear” and “surprise”). Thus, it is important to keep the constraints of a categorization task in mind when discussing generalization of results. Here, observers had to discriminate between seven expressions. This provides a more realistic situation of information uncertainty than discriminating between only two expressions.

Starting from the argument of co-evolution between signalling expressions by the face and their decoding in the brain, we have shown that the 6 basic categories of expression plus neutral form a lowly correlated set of signals. With time-resolved brain data, we have shown that the left and right hemispheres cooperate to decorrelate the expressions for categorization between 140 to 200 ms following stimulus onset.

## Supporting Information

Figure S1 Analysis of the EEG Data: Time Course of the Sensitivity to Combinations of Spatial Frequency Bands (Observer LF, “fear”). Every 4 ms, on electrode OTL and OTR, we represent the combination of the five spatial frequency bands with a binary number (in decimal between 1 and 31) and color code it between white (1) and red (31), see Figure 4 for details. OTR+OTL. To depict the respective contributions of the left and right hemispheres to the encoding of expression features (in the illustration, the left and right eyes), at each time point we added the OTR and OTL classification images and color-coded them (OTR contribution in blue; OTL contribution in red). Contour plots depict the local and/or global spatial extent of the encoding process.
(1.46 MB TIF)

Figure S2 Analysis of the EEG Data: Time Course of the Sensitivity to Combinations of Spatial Frequency Bands (Observer UM, “fear”).
(1.65 MB TIF)

Figure S3 Analysis of the EEG Data: Time Course of the Sensitivity to Combinations of Spatial Frequency Bands (Observer LF, “disgust”).
(1.70 MB TIF)

Figure S4 Analysis of the EEG Data: Time Course of the Sensitivity to Combinations of Spatial Frequency Bands (Observer UM, “disgust”).
(1.65 MB TIF)

Figure S5 Analysis of the EEG Data: Time Course of the Sensitivity to Combinations of Spatial Frequency Bands (Observer LF, collapsed across all seven expressions). For each electrode, we collapsed the time course of sensitivity to the combinations of spatial frequency bands across the seven expressions. OTR+OTL. To depict the respective contributions of the left and right hemispheres to the encoding of expression features, at each time point we added the OTR and OTL classification images across the seven expressions and color-coded them (OTR contribution in blue; OTL contribution in red). Contour plots depict the local and/or global spatial extent of the encoding process.
(1.71 MB TIF)

Figure S6 Analysis of the EEG Data: Time Course of the Sensitivity to Combinations of Spatial Frequency Bands (Observer UM, collapsed across all seven expressions).
(1.72 MB TIF)

Figure S7 Analysis of the EEG Data: Decorrelation of Facial Expressions Between 140 and 200 ms Following Stimulus Onset. A. Backus-Spread Measure of Decorrelation of Facial Expressions (Observers LF). At each time point, for each expression, we transform the classification image (OTR+OTL) into a single high-dimensional vector (of 38×24 image pixels×5 spatial scales of dimensionality). We then cross-correlate the vector for each expression to generate a 7×7 cross-correlation matrix (displayed in the Figure for each time point and electrode OTR, OTL). If the brain aims to individuate expressions, it should decorrelate its representations. In computational terms, the cross-correlation matrices should evolve towards the identity matrix over time (with correlation = 0, in blue, for each pair of expressions; correlation = 1, in red, for self-correlations). Backus-Gilbert Spread (6) measures this distance between the identity and observed matrices. Between 140 and 200 ms following stimulus presentation, the measure identifies three time intervals of decorrelation, represented in a “V”-shaped curve on OTL (red curve) and OTR (blue curve). The black curve reveals the performance of the Model Categorizer which predicts the emotion category from the OTL+OTR classification images plus noise-i.e. from the representation of the expression constructed in the brain. The categorizer was a Winner-Take-All scheme which compared (Pearson correlated) the noisy input with the 70 original stimuli and adjusted noise level, independently for each expression and time point, to maintain classification at 75% correct. The increase of noise level with time (averaged across all 7 expressions) reveals that the representations constructed in the brain are sufficient for behavior and become more robust with time. B. Three Stage of Spatial Frequency Sensivitity in the EEG. The “V” shaped Backus Gilbert curves identify three time intervals of decorrelation (color-coded in green). (a) Within each time interval, we averaged the classification images and color-coded them for the respective contribution of OTR (blue) and OTL (red). These illustrate the time course of the decorrelation process for the facial features of “disgust” and “fear”. (b) The contour plots reveal, in both expressions, that Stage 1 [140–152 ms] contra-laterally encodes the eyes of the face at a local scale. Stage 2 [156–176 ms] zooms out from the local eyes to encode more global face information. Stage 3 [180–200 ms], highlighted with a black box, zooms in to locally and contra-laterally encode the features individuating each expression with maximum decorrelation-i.e. the eyes in “fear” and the corners of the nose in “disgust”.
(1.70 MB TIF)

Figure S8 Analysis of the EEG Data: Decorrelation of Facial Expressions Between 140 and 200 ms Following Stimulus Onset. A. Backus-Spread Measure of Decorrelation of Facial Expressions (Observers UM).
(1.81 MB TIF)
