# Supplementary figures and images for: Transmission of Facial Expressions of Emotion Co-Evolved with Their Efficient Decoding in the Brain: Behavioral and Brain Evidence
Source: PLoS One. 2009 May 20;4(5):e5625. doi: 10.1371/journal.pone.0005625 (PMC2680487; doi:10.1371/journal.pone.0005625)

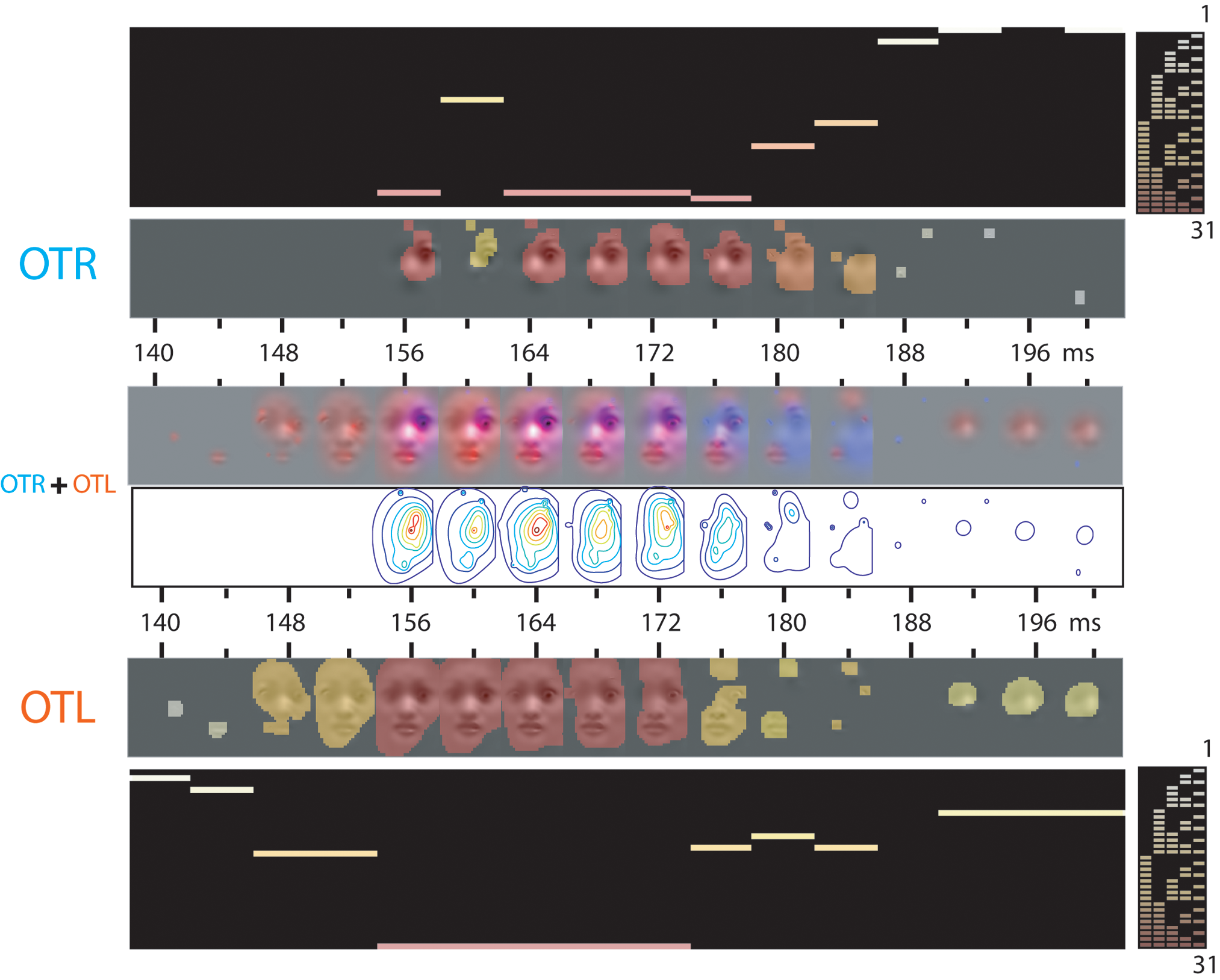

Supplement: Figure S1 — Analysis of the EEG Data: Time Course of the Sensitivity to Combinations of Spatial Frequency Bands (Observer LF, “fear”). Every 4 ms, on electrode OTL and OTR, we represent the combination of the five spatial frequency bands with a binary number (in decimal between 1 and 31) and color code it between white (1) and red (31), see Figure 4 for details. OTR+OTL. To depict the respective contributions of the left and right hemispheres to the encoding of expression features (in the illustration, the left and right eyes), at each time point we added the OTR and OTL classification images and color-coded them (OTR contribution in blue; OTL contribution in red). Contour plots depict the local and/or global spatial extent of the encoding process. (1.46 MB TIF) [file pone.0005625.s001.tif]

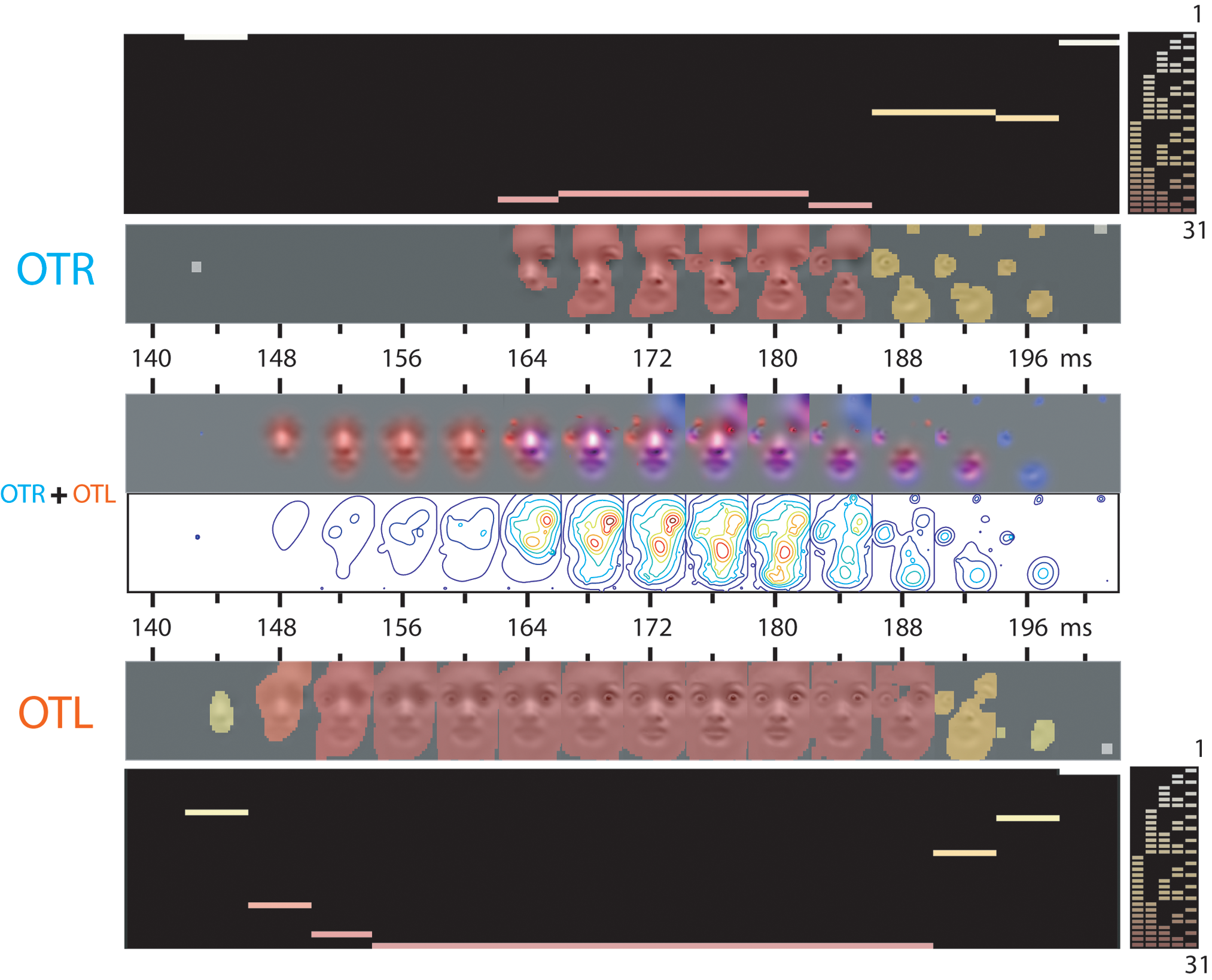

Supplement: Figure S2 — Analysis of the EEG Data: Time Course of the Sensitivity to Combinations of Spatial Frequency Bands (Observer UM, “fear”). (1.65 MB TIF) [file pone.0005625.s002.tif]

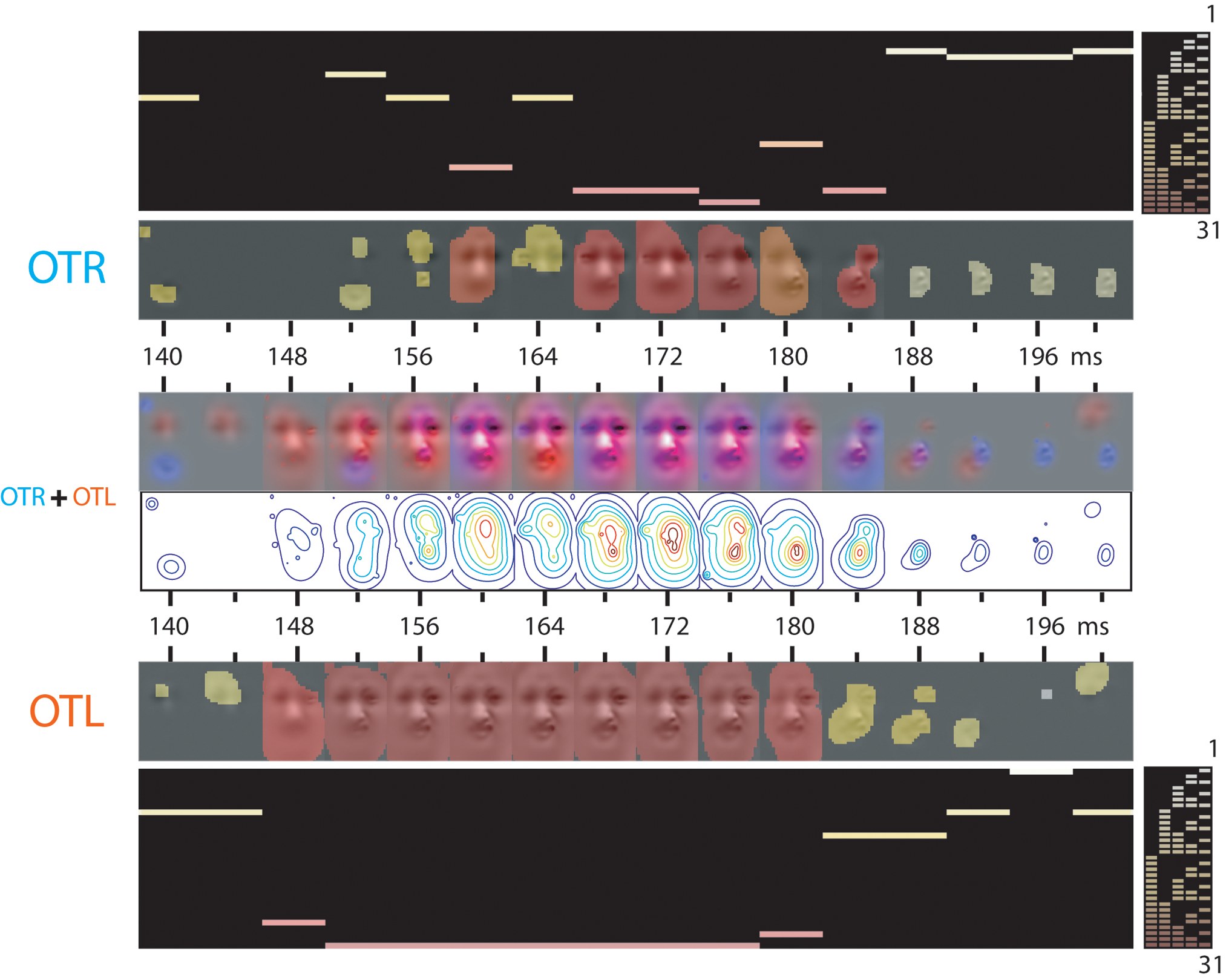

Supplement: Figure S3 — Analysis of the EEG Data: Time Course of the Sensitivity to Combinations of Spatial Frequency Bands (Observer LF, “disgust”). (1.70 MB TIF) [file pone.0005625.s003.tif]

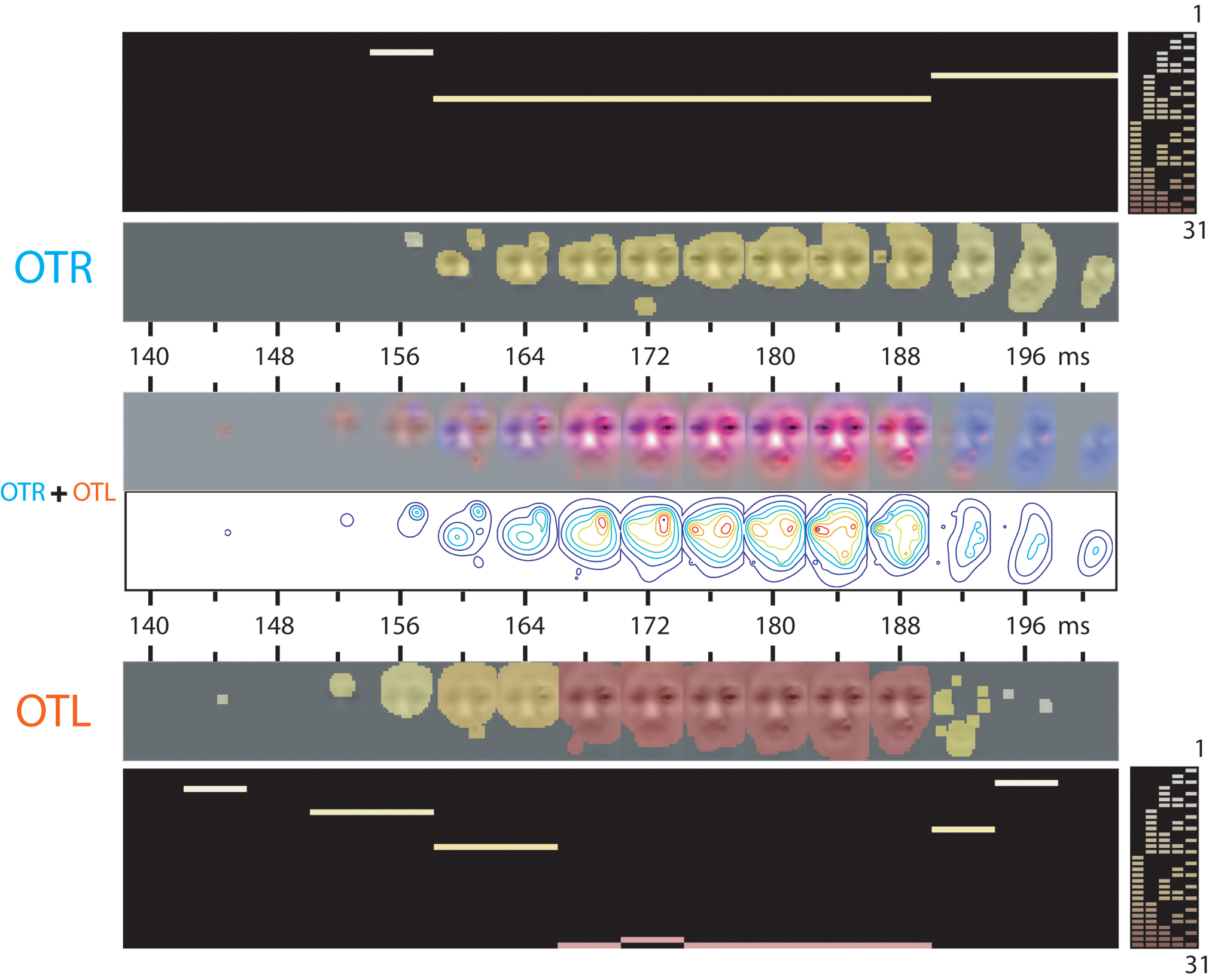

Supplement: Figure S4 — Analysis of the EEG Data: Time Course of the Sensitivity to Combinations of Spatial Frequency Bands (Observer UM, “disgust”). (1.65 MB TIF) [file pone.0005625.s004.tif]

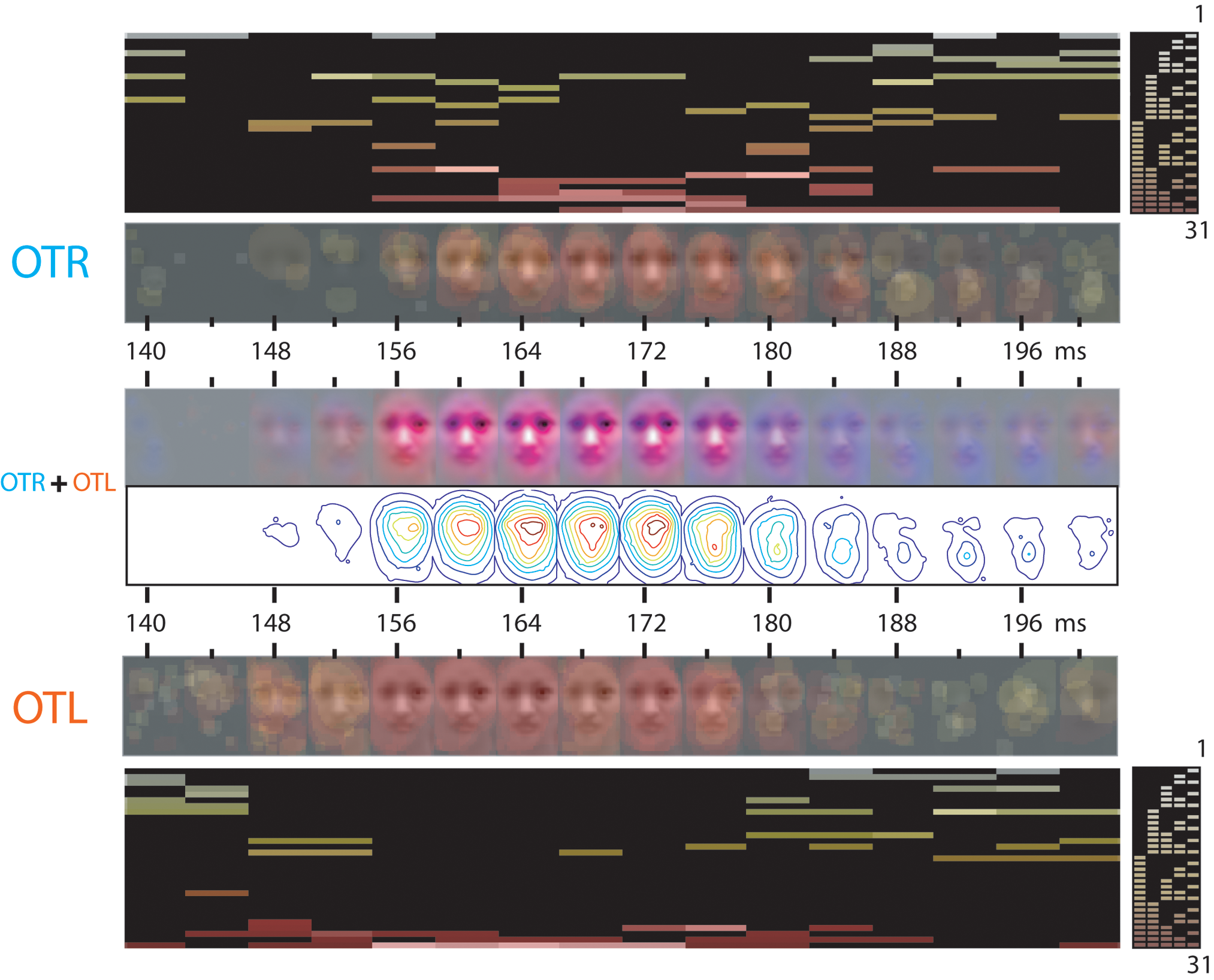

Supplement: Figure S5 — Analysis of the EEG Data: Time Course of the Sensitivity to Combinations of Spatial Frequency Bands (Observer LF, collapsed across all seven expressions). For each electrode, we collapsed the time course of sensitivity to the combinations of spatial frequency bands across the seven expressions. OTR+OTL. To depict the respective contributions of the left and right hemispheres to the encoding of expression features, at each time point we added the OTR and OTL classification images across the seven expressions and color-coded them (OTR contribution in blue; OTL contribution in red). Contour plots depict the local and/or global spatial extent of the encoding process. (1.71 MB TIF) [file pone.0005625.s005.tif]

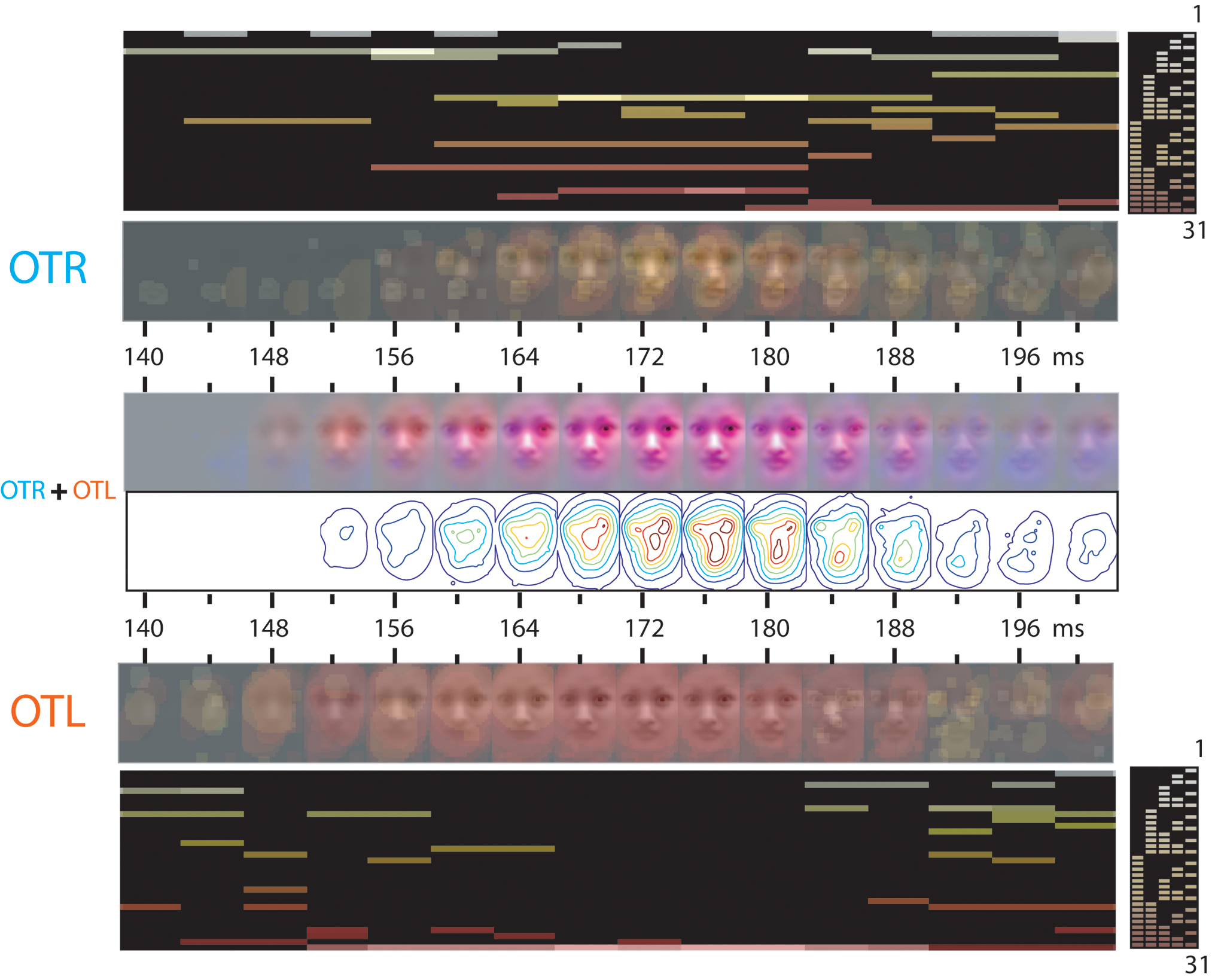

Supplement: Figure S6 — Analysis of the EEG Data: Time Course of the Sensitivity to Combinations of Spatial Frequency Bands (Observer UM, collapsed across all seven expressions). (1.72 MB TIF) [file pone.0005625.s006.tif]

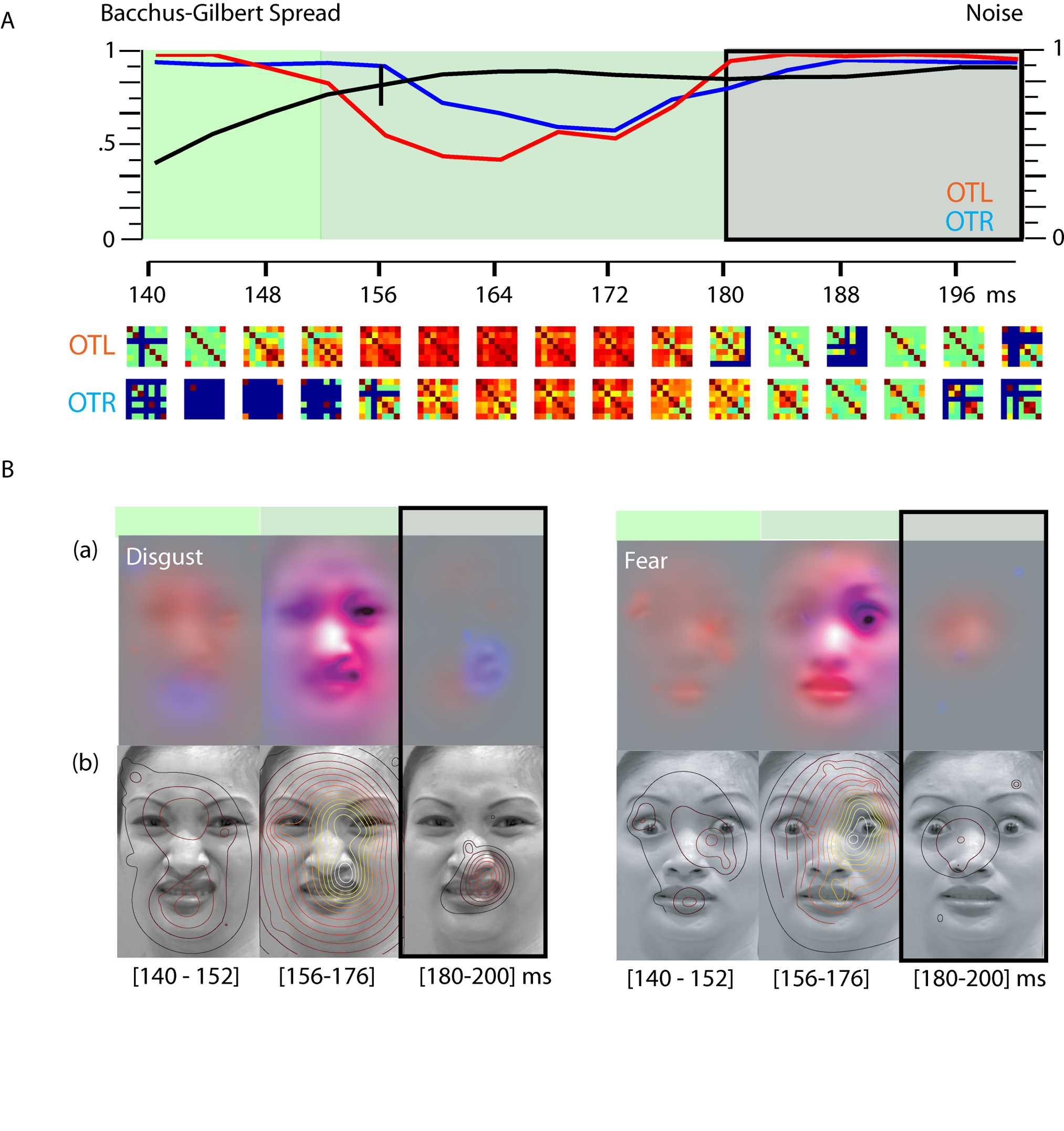

Supplement: Figure S7 — Analysis of the EEG Data: Decorrelation of Facial Expressions Between 140 and 200 ms Following Stimulus Onset. A. Backus-Spread Measure of Decorrelation of Facial Expressions (Observers LF). At each time point, for each expression, we transform the classification image (OTR+OTL) into a single high-dimensional vector (of 38×24 image pixels×5 spatial scales of dimensionality). We then cross-correlate the vector for each expression to generate a 7×7 cross-correlation matrix (displayed in the Figure for each time point and electrode OTR, OTL). If the brain aims to individuate expressions, it should decorrelate its representations. In computational terms, the cross-correlation matrices should evolve towards the identity matrix over time (with correlation = 0, in blue, for each pair of expressions; correlation = 1, in red, for self-correlations). Backus-Gilbert Spread (6) measures this distance between the identity and observed matrices. Between 140 and 200 ms following stimulus presentation, the measure identifies three time intervals of decorrelation, represented in a “V”-shaped curve on OTL (red curve) and OTR (blue curve). The black curve reveals the performance of the Model Categorizer which predicts the emotion category from the OTL+OTR classification images plus noise-i.e. from the representation of the expression constructed in the brain. The categorizer was a Winner-Take-All scheme which compared (Pearson correlated) the noisy input with the 70 original stimuli and adjusted noise level, independently for each expression and time point, to maintain classification at 75% correct. The increase of noise level with time (averaged across all 7 expressions) reveals that the representations constructed in the brain are sufficient for behavior and become more robust with time. B. Three Stage of Spatial Frequency Sensivitity in the EEG. The “V” shaped Backus Gilbert curves identify three time intervals of decorrelation (color-coded in green). (a) Within each time interval, [file pone.0005625.s007.tif]

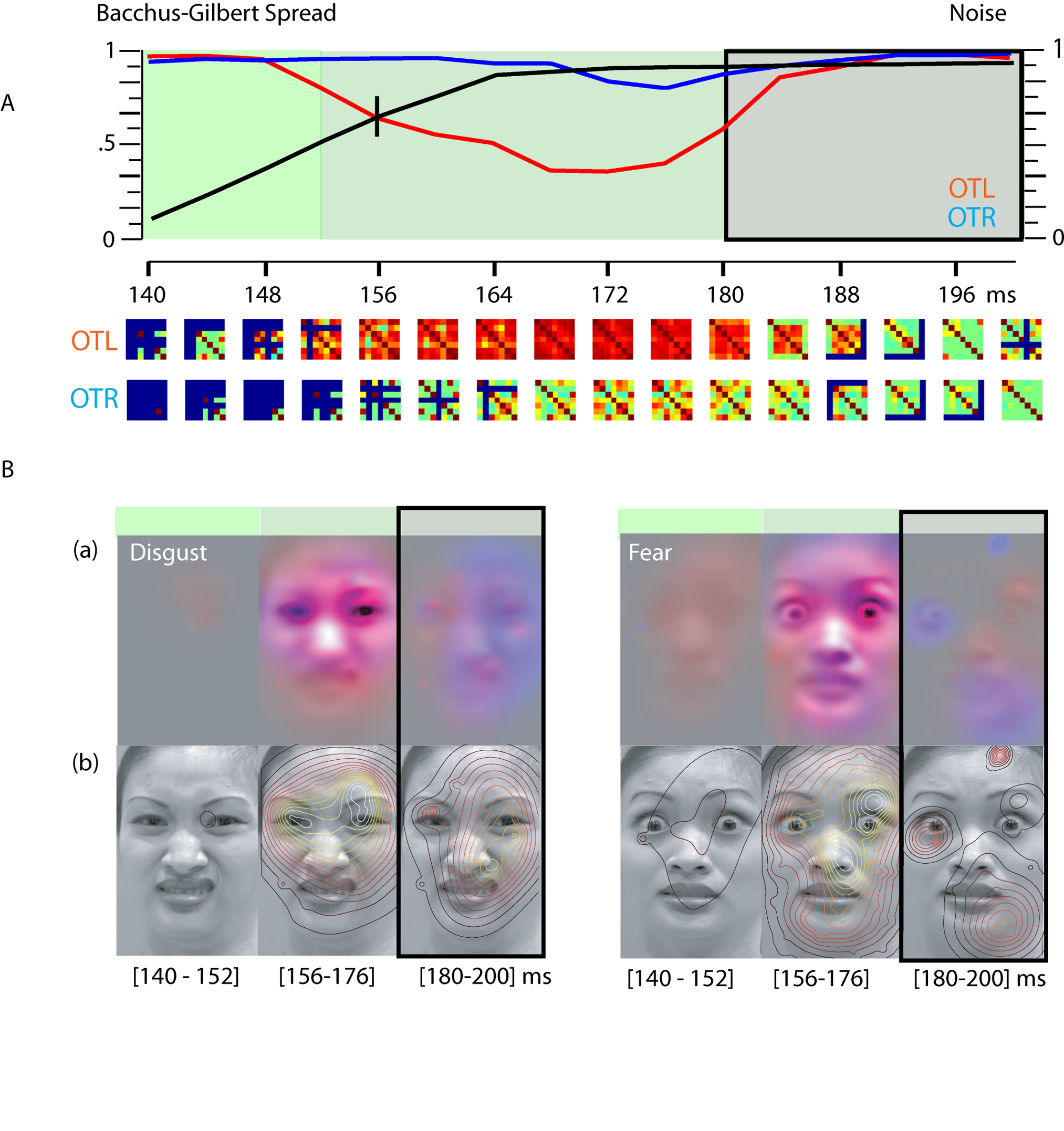

Supplement: Figure S8 — Analysis of the EEG Data: Decorrelation of Facial Expressions Between 140 and 200 ms Following Stimulus Onset. A. Backus-Spread Measure of Decorrelation of Facial Expressions (Observers UM). (1.81 MB TIF) [file pone.0005625.s008.tif]
